# Risk equalization in competitive health insurance markets: Identifying healthy individuals on the basis of multiple‐year low spending

**DOI:** 10.1111/1475-6773.13065

**Published:** 2018-10-16

**Authors:** Frank Eijkenaar, René C. J. A. van Vliet, Richard C. van Kleef

**Affiliations:** ^1^ Erasmus School of Health Policy & Management Erasmus University Rotterdam Rotterdam The Netherlands

**Keywords:** health insurance, health measurement, risk equalization, spending, survey data

## Abstract

**Objective:**

To study the extent to which risk equalization (RE) in competitive health insurance markets can be improved by including an indicator for being healthy.

**Study Setting/Data Sources:**

This study is conducted in the context of the Dutch individual health insurance market. Administrative data on spending and risk characteristics (2011‐2014) for the entire population (N = 16.6 m) as well as health survey data from a large sample (N = 387 k) are used.

**Study Design:**

The indicator for being healthy is low spending in three consecutive prior years. “Low spending” is defined in three ways: belonging to the bottom 60%, 70%, or 80% of the annual spending distribution. Versions of the Dutch RE model 2017 with and without the indicator are compared on individual‐level payment fit and, using the survey data, group‐level payment fit.

**Principal Findings:**

All three alternative models outperform the Dutch RE model 2017. However, significant unpriced risk heterogeneity remains. Compared with the 60% threshold, the 80% threshold comes with a larger improvement in fit but identifies a less selective group.

**Conclusions:**

The performance of the RE model can be improved by adding an indicator for being healthy based on multiple‐year low spending. However, risk‐selection potential remains, warranting high priority to further improvement of RE.

## 1 INTRODUCTION

Unregulated individual health insurance markets tend to risk rating and risk selection, both of which are undesirable from a societal and economic perspective.^1^, ^2^, ^3^, ^4^, ^5^ In practice, therefore, competition in these markets is combined with various regulatory measures to mitigate market failure and promote public objectives. To prevent risk rating, premium‐rate restrictions are typically present. For example, in the individual and small group health insurance markets in the United States, risk rating is limited to age (within rate bands), region, and tobacco use. And in other countries, such as the Netherlands and Germany, premiums are community‐rated per health plan. To prevent risk selection, a system of risk equalization (RE) is usually in place, which compensates insurers for predictable variation in medical spending. Risk selection has been defined as “actions by consumers and insurers to exploit unpriced risk heterogeneity and break pooling arrangements.”^6^ Given premium‐rate restrictions, the goal of RE is to minimize risk‐selection potential by correcting for unpriced risk heterogeneity, that is, by reducing predictable profits and losses on specific subgroups. This paper focuses on mitigating selection potential in the Dutch individual health insurance market.^7^

Over the past decades, RE models in developed countries have evolved from simple demographic models to sophisticated morbidity‐based models, often containing hundreds of risk classes.^8^, ^9^, ^10^, ^11^ However, studies have consistently shown that even state‐of‐the‐art RE models considerably under‐ or overpay specific subgroups in the respective populations, leaving significant selection potential (R. C. Van Kleef, F. Eijkenaar, & R. C. J. A. van Vliet, under review).^10^, ^12^, ^13^, ^14^, ^15^, ^16^, ^17^ Therefore, stakeholders in these markets continue to seek to improve RE, with a strong focus on identifying individuals in *poor health* through the development of new or enhanced morbidity indicators based on prior diagnoses or utilization.^17^, ^18^, ^19^ Importantly, however, there is another side of the issue; risk selection in competitive health insurance markets may also be driven by overcompensated groups of individuals in *good health*, indications of which have been found in several countries, including the United States,^13^, ^15^, ^20^, ^21^ Switzerland,^22^ and the Netherlands.^23^, ^24^, ^25^, ^26^, ^27^

The Dutch RE model 2017, like most such models, includes numerous variables meant to identify sicker, higher‐cost individuals. Specifically, the model contains five morbidity‐based risk adjusters, that is, pharmacy‐based cost groups (PCGs), diagnosis‐based cost groups (DCGs), durable medical equipment groups (DMEGs), physiotherapy diagnosis groups (PDGs), and multiple‐year high‐spending (MYHS) groups.^11^ About 27% of the population is flagged by one or more of these variables. In this paper, the focus is on the complementary group of individuals not flagged by a morbidity variable (73% of the population) and who are thus *implicitly* designated as healthy. However, this group is likely to be heterogeneous in terms of health and spending, implying the existence of unpriced risk heterogeneity (and thus selection potential) within this group. The reason is 2‐fold. First, not all chronic conditions involving predictable, above‐average spending are captured by the morbidity variables. Second, the morbidity variables may not flag individuals in *moderate* health (eg, those who are just developing a chronic illness). For example, individuals are only classified in a PCG if they meet a threshold of 180 defined daily doses of the relevant drugs per year. Indeed, data confirm that many individuals with chronic illnesses are missed: About 50% of the Dutch population is considered to be chronically ill according to International Classification of Primary Care codes, with “chronic illness” defined as an illness without any prospect of full recovery.^28^ The result is that within the group without a morbidity flag, individuals in moderate or poor health are undercompensated while those in good health are overcompensated.

Using administrative data on medical spending and risk characteristics over a 4‐year period for the entire Dutch population (N = 16.6 m) as well as data from a health survey conducted among a large sample (N = 387 k) of that population, this paper investigates to what extent the Dutch RE model can be improved by *explicitly* identifying individuals likely to be healthy given their low prior spending levels. Specifically, our goal is 2‐fold: (a) identifying healthy individuals on the basis of multiple‐year low spending and (b) examining the impact of adding an indicator for “being healthy” into alternative versions of the RE model on payment fit (ie, the extent to which insurers’ revenues from RE match the insurance claims), both at the individual level and at the level of specific subgroups derived from the health survey. In addition, models are evaluated on their potential impact on cost‐containment incentives, which is relevant here since adding the indicator for “being healthy” creates a link between (prior) spending and (future) RE payment.

The paper proceeds as follows. After a brief description of the Dutch health insurance system and RE model, the data and methodology are explained. Next, the main results are presented, followed by a discussion of the conclusions and the policy implications.

## 2 THE DUTCH HEALTH INSURANCE SYSTEM AND RISK EQUALIZATION MODEL

The Dutch health insurance system is based on Enthoven's model of regulated competition, combining competition with regulation to promote efficiency and protect public objectives such as accessibility and affordability.^7^, ^29^, ^30^ Competition is driven by free consumer choice of health plan. Within bounds set by regulation, plans can differ in terms of provider network, coverage of out‐of‐network spending, and cost‐sharing options. Regulation includes an individual insurance mandate, annual open enrollment, a standardized benefit package, community‐rated premiums, and RE among insurers.^11^

In 2017, the Dutch RE system consisted of three different models: for somatic care (ie, primary care, hospital care, and pharmaceutical care), for mental care, and for the out‐of‐pocket payments due to the mandatory deductible (385 euro per person in 2017). In this paper, the focus is exclusively on the somatic model, pertaining to about 90% of spending covered by the standardized benefits package. Coefficients of the RE model for year t are estimated by an individual‐level ordinary least squares regression of spending in year *t*‐3 on risk characteristics from year *t*‐3 or before (depending on the risk adjuster). Prior to estimation, several modifications are applied to make the lagged data representative for year *t*.^11^

The somatic RE model 2017 contains almost 200 risk classes, including 40 age/gender classes, 33 PCGs based on the prior use of drugs prescribed for chronic illnesses, 15 DCGs based on diagnoses of hospital treatment in the prior year, 10 DMEGs based on prior use of durable medical equipment, 4 PDGs based on diagnoses of physiotherapy in the prior year, and 7 MYHS groups based on high spending in the prior 3 years. With the exception of the PCGs and PDGs, the Dutch model does not include information on primary care diagnoses/utilization because the required information is not available for the whole population. This is also an important reason for why we use low spending as an indicator for “being healthy.”

In 2017, the RE model also contained two risk adjusters based on spending on home care and on geriatric rehabilitation care in the prior year. However, both adjusters are excluded here as the latter has recently been removed and the former will probably be replaced in the RE model of 2019.

The introduction of spending‐based risk adjusters in the Dutch RE model is primarily a result of the importance being attached by relevant stakeholders to mitigating risk‐selection potential. There is strong preference for realizing this goal via ex ante compensation based on medically/clinically informed adjusters based on diagnoses and/or utilization linked to chronic illness. But as long as selection potential remains and the data required for developing such adjusters are not available, spending‐based adjusters have been used in the Dutch model since they can be effective in reducing unpriced risk heterogeneity. However, given the direct link between spending and RE payment, these adjusters reduce incentives for cost containment, implying a trade‐off. In the opinion of the Dutch government (and other stakeholders), the reduction in selection potential outweighs the reduction in incentives for cost containment. The government has stated, however, that spending‐based risk adjusters are a second‐best solution and will be replaced as soon as better alternatives become available.^31^

## 3 DATA AND METHODS

### 3.1 Administrative data and health survey data

Two datasets are available for this study. First, we use administrative data on medical spending and risk characteristics for the entire Dutch population (N = 16.6 million) for a 4‐year period (2011‐2014). These data were those actually used for calculating the coefficients of the RE models for the years 2014‐2017, respectively. We use these data to identify individuals likely to be healthy (in absolute sense) based on low prior spending, replicate the RE model 2017, and compare the individual‐level fit of alternative versions of that model. We also use these data to simulate the impact of including an indicator for “being healthy” on insurers’ cost‐containment incentives.

In addition, models are compared on group‐level fit. This is a common approach to quantifying unpriced risk heterogeneity in health insurance markets.^32^ This approach, however, requires health information not included in the RE model. Therefore, we use a second dataset based on a health survey conducted among a large sample (N = 387 195) of the population in 2012. These data contain rich information on self‐reported general health and chronic conditions,^33^ which can be used to define subgroups with an over‐ or underrepresentation of people in poor health. In turn, for each of these subgroups, the mean actual spending can be compared with the mean spending predicted by alternative RE models, providing an indication of each model's group‐level fit.

The survey sample is not representative of the entire population in three ways. First, individuals living in an institution for long‐term care are not included. Second, the sample only includes individuals of 19 years or over (on September 1, 2012). Consequently, results on group‐level fit are conditional on the remaining (adult) sample. Third, the remaining sample was not drawn randomly. To correct for nonrandom sampling regarding several factors (eg, age, gender, ethnicity, and income), we reweighted the sample using weights supplied by Statistics Netherlands.

### 3.2 Identifying healthy individuals on the basis of multiple‐year low spending

Our first objective was to identify individuals likely to be healthy in absolute sense based on multiple‐year low spending (MYLS). To our knowledge, low‐spending indicators have not been used previously in a RE model, so our choice of thresholds (ie, the place in the spending distribution) and of the number of years necessary to be designated as healthy was based on our own judgment rather than previous empirical research. We chose a period of three consecutive prior years as a relatively high bar for consistency of low spending. Low spending in one or two prior years could easily be a result of (more or less) random spread of insurance claims across calendar years or temporary upswings in health. In addition, 3 years corresponds to the definition of the current MYHS risk adjuster,^34^ contributing to within‐model consistency. Nevertheless, we also investigated the potential added value of using two instead of three‐year low spending.

Regarding the place in the spending distribution, we were guided by the finding that an estimated 50% of the Dutch population is not chronically ill (Volksgezondheidenzorg.info 2018a). We first determined (by trial and error) the spending threshold in each of the three prior years (2011‐2013) that is required to yield 50% of the population in 2014. This resulted in a percentile of 69.7%, which we rounded to 70%, corresponding to a spending threshold of approximately 1100 euro (about half of overall mean spending in 2014). According to this definition, individuals are designated as “healthy” in 2014 if they belong to the bottom 70% of the spending distribution in each of the three prior years 2011‐2013. To determine the sensitivity of our results for the choice of spending threshold, we also examine two alternative thresholds, 60% and 80%.

### 3.3 Payment fit and cost‐containment incentives

Separately for each of the three spending thresholds, we constructed an indicator for “being healthy” and incorporated it into the RE model 2017. The resulting models are compared on payment fit at the individual level and subgroup level. Individual‐level fit is assessed using the *R*‐squared, Cumming's prediction measure,^35^ and the mean absolute prediction error.

To assess models’ group‐level fit, we first merged the actual spending and the predicted spending (based on each of the four models, which were all estimated on the administrative data containing all 16.6 million individuals) in 2014 with the health survey data using an anonymized individual‐level identification key. Next, using the information in the survey data, we defined 28 subgroups that are overrepresented by individuals in either poor or in good health, and calculated the mean per person under/overcompensation for each of these subgroups by subtracting the mean actual spending from the mean predicted spending, separately for each model. Assessing payment fit in this way is considered an adequate method for quantifying unpriced risk heterogeneity in health insurance markets, but is often not feasible in practice due to a lack of “external” health information that is not included in the RE model.^32^ We circumvent this problem by merging the administrative data with rich health information from a survey conducted among a large sample.

Almost 98% of the survey respondents matched successfully with the administrative data of 2014. Main reasons for an unsuccessful match are death and migration in 2012 or 2013. Table 1 presents information on actual and predicted spending for adults in the administrative data and survey respondents matching with these data. Based on these results, the sample seems slightly healthier than the total adult population. Previous papers using the same data have presented more detailed comparisons of both groups and similarly concluded that the sample is slightly healthier (R. C. Van Kleef, F. Eijkenaar, & R. C. J. A. van Vliet, under review).^19^, ^36^ In this study, this results in a small overcompensation on the sample of 46 euro. We did not correct for this overcompensation because (a) we do not know how it is distributed over specific groups, and (b) our goal is to assess the relative performance of alternative RE models rather than these models’ absolute performance. Nonetheless, we assessed the impact of recalibrating the survey data, such that for each model, the mean predicted spending equaled the mean actual spending. This did not alter our conclusions since the relative differences among models (which was our main focus) did not change.

**Table 1:** Mean (predicted) spending and overcompensation for adult individuals in the administrative data (2014) and for survey respondents (2012) who successfully match with the administrative data

	Adult individuals in administrative data (2014)	Survey respondents (2012) who match with administrative data (2014)
Weighted number of individuals^a^	12 659 298	375 742
Mean actual spending (€)^b^	2590	2561*
Mean predicted spending (€)	2587	2607*
Mean under/overcompensation (€)^c^	−3	46*

a The number of individuals who were at least 19 y old on September 1, 2012 weighted by the duration of enrollment in 2014. The total weighted number of individuals in the administrative data of 2014 is 16 607 620, with mean spending of €2227.

b Spending refers to spending on curative somatic care (eg, primary care, pharmaceutical care, and hospital care) covered by the benefits package of 2017 (cost/price level of 2014).

c Defined as the mean predicted spending in 2014 (based on the RE model 2017) minus the mean actual spending in 2014.

^*^Statistically significantly different from the corresponding number for the administrative data, based on a two‐sided *t* test (*P* < 0.05).

Since adding the indicator for “being healthy” creates a link between (prior) spending and (future) RE payments, we also evaluate models on the potential impact on insurers’ cost‐containment incentives by (a) qualitatively assessing the possibilities for strategic behavior (ie, stimulating and/or not preventing individuals from exceeding the spending threshold) and (b) simulating the effect on RE payments of a small or medium‐sized insurer letting its total insurance claims increase generically by 1% in the prior year (2013). In spirit, the latter relates to the “power measure” developed by Geruso and McGuire,^37^ with the main difference that we examine the marginal change in RE payments due to a marginal change in *claims* (instead of *utilization*). This measure describes how regulators compensate spending at the margin, or how RE impacts insurers’ marginal incentive to contain costs. In the Netherlands as well as in many other countries, this is relevant as insurers are in the position to influence consumers’ and providers’ utilization decisions.^38^ In general, competing insurers may seek to encourage utilization that increases the marginal benefit resulting from higher RE payments more than the marginal cost resulting from higher utilization.^37^

## 4 RESULTS

### 4.1 Identifying healthy individuals on the basis of multiple‐year low spending

Table 2 shows descriptive statistics for the three groups identified based on three‐year spending below 60%, 70%, or 80%. Unsurprisingly, the mean spending threshold (in euros), the size of the group identified, and the mean spending increase with higher thresholds. The opposite holds for the mean overcompensation, which reduces from 231 euro for the 60% threshold to 185 euro for the 80% threshold. Thus, in terms of mean overcompensation and spending, the 60% threshold yields the most selective group. However, the *total* overcompensation (ie, taking the size of the group into account) is considerably higher for the higher thresholds and highest based on the 80% threshold.

**Table 2:** Characteristics of three groups of individuals designated as “healthy” on the basis of multiple‐year low spending (2011‐2013) using three different spending thresholds

Group identified based on	Mean spending threshold (€)	Weighted % of individuals in 2014 (%)^a^	Mean spending in 2014 (€)^b^	Mean overcompensation in 2014 (€)^c^	Total overcompensation in 2014 (millions of €)^d^
Three‐year spending <60%	662	41.0	717	231	1571
Three‐year spending <70%	1081	53.2	796	208	1836
Three‐year spending <80%	1916	66.5	943	185	2040

a The number of individuals weighted by the duration of enrollment in 2014 as a percentage of the total population in 2014 (16 607 620).

b Spending refers to spending on curative somatic care (eg, primary care, pharmaceutical care, and hospital care) covered by the benefits package of 2017, at the cost/price level of 2014. The overall mean total spending in 2014 equals €2227.

c Defined as the mean predicted spending in 2014 (based on the Dutch RE model 2017) minus the mean actual spending in 2014.

d Defined as the mean overcompensation in 2014 multiplied by the weighted number of individuals in the relevant group.

An additional analysis (data not shown) revealed that increasing the threshold further (eg, to 81%) would not yield an even higher total overcompensation: While the 1% group with three‐year spending below 80% but *not* below 79% is still overcompensated, the 1% group with three‐year spending below 81% but *not* below 80% is undercompensated.

We also examined the modality “low spending in two out of three prior years” and found that the mean overcompensation in 2014 for the resulting group (comprising 50% of the whole population) is almost 10% *lower* than the group identified based on three‐year spending below 70%. Since this modality thus results in a less selective group and involves much lower spending thresholds (around 400 euro, which will probably be considered problematic in the light of insurers’ cost‐containment incentives), we did not investigate this modality further.

### 4.2 Payment fit

This section compares the fit of the RE model 2017 and three alternative versions of that model. The alternative versions all include a MYLS‐based indicator but use different thresholds to assign individuals to that indicator. Note that adding such an indicator implies that the group not classified in the existing “multiple‐year high spending” (MYHS) adjuster is essentially split into two, that is, (a) a group with MYLS and (b) a group without MYLS *and* without MYHS.

The first three rows of Table 3 show that alternative models 2‐4 clearly outperform model 1 on individual‐level fit. Though statistically significant, the difference in fit among models 2‐4 is small: Compared to model 1, the improvement in Cumming's prediction measure (+0.6 to +0.8 percentage point) is relatively large, while the *R*‐squared improves only marginally.

**Table 3:** Individual‐level payment fit and estimated coefficients for relevant risk classes for four versions of the Dutch RE model 2017

	Model 1 = RE model 2017	Model 2 = model 1 + a risk class for 3‐y spending <60%	Model 3 = model 1 + a risk class for 3‐y spending <70%	Model 4 = model 1 + a risk class for 3‐y spending <80%
Fit statistic^a^				
*R*‐squared × 100%	28.7	28.8	28.8	28.8
Cumming's prediction measure × 100%	28.1	28.7	28.9	28.9
Mean absolute prediction error (€)	2021	2002	1999	1998
Estimated coefficients (€)				
No MYHS	−287	‐	‐	‐
Three‐year spending <60%	‐	−576	‐	‐
≥One‐year spending ≥60% and no MYHS	‐	−87	‐	‐
Three‐year spending <70%	‐	‐	−565	‐
≥One‐year spending ≥70% and no MYHS	‐	‐	32	‐
Three‐year spending <80%	‐	‐	‐	−543
≥One‐year spending ≥80% and no MYHS	‐	‐	‐	249

MYHS, multiple‐year high spending.

a *R*‐squared = proportion explained variance = 1 − (sum of squared residuals/total sum of squared spending differences). Cumming's prediction measure = 1 − (sum of absolute residuals/total sum of absolute spending differences). Mean absolute prediction error = sum of absolute residuals divided by the weighted number of individuals.

Table 3 also shows the estimated coefficients for relevant risk classes. The coefficient of interest in model 1 is that of the class “No MYHS”: −287 euro. In models 2‐4, this class is split into two, explicitly distinguishing between individuals with MYLS, and those without MYLS *and* without MYHS. In model 3, for example, this yields a coefficient of −565 for the group with MYLS and a coefficient of 32 euro for the complementary group. As the threshold goes up, the latter group becomes more selective and the accompanying coefficient higher, up to 249 euro in model 4. This illustrates how adding an indicator based on MYLS can improve compensation for both individuals in good health and individuals in moderate or poor health.

As a result of adding a MYLS‐based indicator, the overcompensation on the groups designated as healthy (see Table 2) naturally reduces to zero. Table 4 compares the models on payment fit for 28 other groups, derived from the health survey data. Based on the overall figure shown at the bottom of the table, each of the alternative models improves upon the fit of the RE model 2017, with model 4 yielding the largest improvement (−18% compared to model 1).

**Table 4:** Mean spending and under/overcompensation in 2014 based on four versions of the Dutch RE model 2017 for 28 subgroups defined based on health survey data of 2012

Subgroup	Estimated size vs total adult population (%)	Mean spending in 2014 (€) c	Mean under/overcompensation in 2014 (€)^a^^,^ b			
			Model 1 = RE model 2017	Model 2 = model 1 + 3‐y spending <60%	Model 3 = model 1 + 3‐y spending <70%	Model 4 = model 1 + 3‐y spending <80%
General health (mutually exclusive)						
Good or very good	76.9	1611	140**	135**	131**	130**
Fair, poor, or very poor	23.1	5653	−275**	−239**	−228**	−219**
(Co)morbidity (mutually exclusive; past year)						
No self‐reported condition	39.7	1102	175**	143**	141**	144**
1 self‐reported condition	28.3	2277	103**	121**	117**	113**
2 self‐reported conditions	14.9	3250	−40	2	4	−4
3 self‐reported conditions	7.6	4396	−108*	−62	−56	−50
≥4 self‐reported conditions	9.5	6662	−428**	−400**	−383**	−365**
General health * (Co)morbidity						
Good or very good general health and no self‐reported condition	32.7	1005	197**	157**	155**	159**
Self‐reported condition (ever)						
Diabetes	6.0	7024	33	48	51	49
Stroke	2.8	7232	−374**	−356**	−345**	−322**
Acute myocardial infarction	3.1	7656	−160	−147	−138	−119
Cancer	6.6	6298	−146*	−108	−99	−84
Self‐reported condition (past year)						
Heart condition	2.1	8818	−483*	−466**	−450**	−412**
Migraine and severe headache	16.9	2384	−91**	−66**	−65**	−70**
Hypertension	18.2	4606	−92**	−60	−56	−56
Peripheral artery disease	2.7	8008	−764**	−751**	−741**	−725**
Asthma/chronic bronchitis/emphysema	8.7	4817	−98*	−66	−61	−66
Psoriasis	3.0	3781	−83	−51	−47	−55
Chronic eczema	5.4	2787	−89	−63	−68	−72
Dizziness with falling	4.3	5567	−481**	−451**	−440**	−428**
Severe/recurrent disease of intestines	4.8	5035	−329**	−274**	−258**	−249**
Incontinence	6.8	5590	−381**	−358**	−348**	−334**
Arthrosis or arthritis of hip(s)/knee(s)	14.7	5054	−220**	−189**	−178**	−164**
Chronic inflammation of joints	5.6	6075	−312**	−283**	−277**	−270**
Severe/recurrent condition of back	11.0	4297	−331**	−295**	−287**	−277**
Severe/recurrent condition of neck/shoulders	10.6	3912	−245**	−207**	−200**	−195**
Severe/recurrent condition of elbow/wrist/hand	6.9	4606	−294**	−254**	−244**	−231**
Other self‐reported condition	15.1	4796	−157**	−106**	−92*	−85*
Overall absolute mean (% reduction vs model 1)^d^			184	160 (−13%)	154 (−16%)	151 (−18%)

a Defined as the mean predicted spending for a group in 2014 (based on the relevant RE model) minus the mean actual spending for that group in 2014. Thus, a negative amount indicates an undercompensation and a positive amount an overcompensation.

b Note that the weighted mean of the under/overcompensations of mutually exclusive groups does not equal 0 due to the fact that the overall mean (predicted) spending in the survey data differs slightly from the overall mean (predicted) spending in the administrative data.

c Spending refers to the total curative somatic spending in relation to the basic health insurance package of 2017 (cost/price level of 2014). The mean spending in the sample as a whole equals 2561 euro (see Table 1).

d Calculated as the overall mean of the absolute values of the under/overcompensations, weighted by the size of the subgroups.

^*^Statistically significantly different from 0 based on a two‐sided *t* test (*P* < 0.05).

^**^Statistically significantly different from 0 based on a two‐sided *t* test (*P* < 0.01).

Adding an indicator based on MYLS improves the compensation for 23 of the 25 groups with an overrepresentation of individuals in *poor* health, the exceptions being the groups “1 self‐reported chronic condition (past year)” and “diabetes (ever).” Interestingly, both are *over*compensated by the RE model 2017, and adding the indicator apparently increases this overcompensation somewhat, though for “diabetes (ever)” the overcompensation is not statistically significant for any model.

The results for the three remaining groups are of particular interest as they are overrepresented by individuals in *good* health, that is, the 77% individuals assessing their own health as good or very good, the 40% individuals reporting no chronic condition over the past year, and the 33% individuals that can be found in both of these groups. For these three groups, the improvement in fit is relatively modest: The overcompensation drops by maximally 7% (model 4), 19% (model 3), and 21% (model 3), respectively.

To further investigate this, we calculated the number of survey respondents in the relevant group as a percentage of the *total* number of survey respondents with MYLS, separately for each threshold. Conversely, we calculated the number of survey respondents with MYLS as a percentage of the *total* respondents comprising each group. The results are shown in Table 5 and suggest that (a) the group identified based on MYLS contains relatively many individuals in poor self‐reported health (see the top three rows) and (b) many of the respondents comprising the three subgroups are not designated as “healthy” based on MYLS (bottom three rows). Both could explain the fairly modest improvement in fit for these three subgroups.

**Table 5:** Heterogeneity of three groups of individuals designated as “healthy” on the basis of multiple‐year low spending (2011‐2013) using three different spending thresholds

	Survey respondents as a percentage of		
	All respondents with 3‐y spending <60%	All respondents with 3‐y spending <70%	All respondents with 3‐y spending <80%
(Very) good self‐reported health^a^	92%	90%	87%
No self‐reported condition^a^	60%	56%	51%
(Very) good self‐reported health and no self‐reported condition^a^	51%	47%	42%
	**All respondents with (very) good self‐reported health** a	**All respondents with no self‐reported condition** a	**All respondents with (very) good self‐reported health and no self‐reported condition** a
Three‐year spending below 60%	44%	56%	57%
Three‐year spending below 70%	56%	67%	69%
Three‐year spending below 80%	69%	78%	79%

a Subgroup defined based on the health survey data.

### 4.3 Cost‐containment incentives

A potential drawback of an indicator based on MYLS is that it could mitigate insurers’ incentives for cost containment. If an individual exceeds the relevant spending threshold at least once in the three prior years, based on the coefficients shown in Table 3, this implies an extra RE payment for his/her insurer in the current year of around 560 euro (relative to the situation in which the individual would stay below the threshold in the entire three‐year period). This could stimulate insurers to (a) behave strategically (ie, not preventing individuals from slightly exceeding the relevant spending threshold) and/or (b) refrain from enacting specific cost‐containment strategies. However, it is unlikely that insurers will actually act on these incentives in practice as the potential benefits are either highly uncertain or unlikely to be worth the additional costs.

Regarding the former, using a percentage instead of an absolute threshold makes the potential benefits of strategic behavior uncertain as they depend on the actions of other insurers. In addition, regarding individuals who already remained below the threshold for 2 years, close to the end of the third year, insurers would have to determine whether these individuals will stay under the threshold again and if so, to take action. But at that moment, claims for ongoing treatments and for treatments that have yet to start are not available. Furthermore, even if insurers would succeed in pushing certain individuals over the spending threshold (and these individuals do not switch to other insurers, which about 25% of the individuals identified based on the 70% threshold did in the three prior years), the resulting additional RE payments may well be one‐off as individuals could require an expensive treatment in the next year. Moreover, the utilization required to push individuals over the threshold generates additional claims, reducing net benefits.

To illustrate that the benefits from not enacting specific cost‐containment strategies are unlikely to be worth the costs, we calculated the additional RE payments (based on each of the three alternative RE models) that would be generated if a medium‐sized insurer would let his insurance claims increase generically by 1% in the prior year (2013). In our data, this would imply a 370 million euro increase in claims, which for the 60%, 70%, and 80% threshold would result in a 21, 23, and 27 million euro increase in RE payment as a result of a 0.26%, 0.23%, and 0.22% increase in enrollees exceeding the threshold, respectively. In other words, to gain 1 euro in additional payments in the current year, depending on the threshold insurers must “invest” 18 euro (=370/21), 16 euro (=370/23), or 14 euro (=370/27) in the prior year.

## 5 DISCUSSION

In the sophisticated Dutch RE model 2017, about 27% of the population is flagged by a morbidity‐based risk adjuster. Although the complementary group is *implicitly* designated as healthy, this group is heterogeneous in terms of health and spending. Consequently, risk‐selection potential exists in this group, which is undesirable in view of the unfavorable effects of risk selection and the finding that risk selection has also been driven by overcompensated groups of individuals in good health.^13^, ^15^, ^20^, ^21^, ^22^, ^23^, ^24^, ^25^, ^26^, ^27^ This paper investigated the extent to which the fit of the RE model can be improved by *explicitly* identifying individuals likely to be healthy (in absolute sense) using an indicator‐based multiple‐year low spending. Based on three definitions of “low spending,” three alternative versions of the Dutch RE model 2017 were constructed and subsequently compared. We came to four main conclusions. First, all three alternative models that contain an indicator for “being healthy” outperform the RE model 2017 in terms of payment fit. This conclusion holds both at the individual level and at the level of 28 specific subgroups from a health survey and suggests that adding an indicator for “being healthy” would reduce risk‐selection potential in the Dutch health insurance market.

Regarding group‐level fit, compensation not only improves for groups that are overrepresented by individuals in good health, but also for almost all groups comprising individuals with a self‐reported chronic condition. The explanation is that the reduced RE payment for the healthy implies a higher RE payment for their unhealthy counterparts. For three large groups of individuals in good self‐reported health, however, the improvement in fit is modest, indicating that the groups designated as healthy based on low prior spending contain relatively many people with a poor self‐reported health, and vice versa. Indeed, an additional analysis on the survey sample showed that the identified groups of healthy individuals are to some extent heterogeneous, suggesting that MYLS is not entirely measuring what it is intended to measure, which is “being healthy.” Unfortunately, with the data available, it was not possible to conduct further validations and determine what the MYLS indicator is actually measuring in addition to good health.

In theory, compensation for the three subgroups that are overrepresented by individuals in good health can be improved by explicitly including the relevant survey variables into the RE model. In practice, however, this is problematic as these data are not available for the whole population. If incorporating this information in the RE model is nonetheless considered desirable, other estimation methods, such as constrained least squares regression, may be required (A. A. Withagen‐Koster, R. C. van Kleef, & F. Eijkenaar, To be submitted for publication).^39^

The second conclusion is that although differences are small, the improvement in fit increases with the share of individuals designated as healthy. As compared to the 60% and 70% thresholds, the 80% threshold discriminates more between the designated group and the complementary group. Also, the *total* overcompensation (ie, taking account of the size of the group) is highest under the 80% threshold. In terms of the *mean* overcompensation, however, the 80% threshold yields a less selective group as compared to the groups identified based on the two lower thresholds.

We did not differentiate the spending threshold(s) for potentially relevant enrollee characteristics (such as yes/no morbidity classification). The reason is that our goal was to identify individuals who are healthy in *absolute* sense (and not in *relative* sense, eg, individuals with relatively low‐spending levels within the group with a morbidity classification), which was informed by the fact that selection actions by Dutch insurers over the past decade have mainly been targeted at those types of individuals. Nonetheless, we acknowledge that a differentiated threshold might be able to further reduce unpriced risk heterogeneity within the group of chronically ill individuals, which will reduce incentives to attract the relatively healthy individuals within this group (and to deter the relatively unhealthy). We believe this is an interesting topic for follow‐up research.

The third conclusion is that insurers’ incentives for cost containment are unlikely to be materially affected by adding an indicator based on MYLS. Concerns about these incentives being reduced might seem justified since a link is created between spending and payments, which could stimulate insurers to behave strategically and/or to refrain from enacting specific cost‐containment strategies. However, our results indicate that the likelihood of insurers acting on these incentives will be low in practice because, at least in the Dutch context, the benefits are either uncertain or unlikely to be worth the costs. It must be noted, however, that our data did not permit explicit modeling of the likelihood and impact of strategic behavior under different MYLS modalities, which may have been more informative. Assessing how and to what extent insurers in general respond to the introduction of spending‐based risk adjusters in RE models is an important topic for future research.

A related limitation is that although the goal of the Dutch RE model is to compensate insurers for predictable, *health‐related* variation in spending, introducing a MYLS variable to the model would undoubtedly result in insurers also being partly compensated for variation related to factors other than health, such as enrollees’ proclivity to consume health care and insurers’ efficiency. In general, this also holds for the other diagnosis‐/utilization‐based risk adjusters in the model and inevitably involves a trade‐off with no obvious escape.

The final conclusion is that regardless of the model or threshold used, unpriced risk heterogeneity in the Dutch individual health insurance market remains considerable. This is consistent with findings from recent other studies conducted in the Dutch context (R. C. Van Kleef, F. Eijkenaar, & R. C. J. A. van Vliet, under review)^19^, ^36^ as well as in other countries with sophisticated morbidity‐based RE models.^10^, ^12^, ^13^, ^14^, ^15^, ^16^, ^17^ An interesting direction for further research is to investigate the extent to which remaining risk‐selection potential in these countries can be meaningfully mitigated further by introducing new risk adjusters or that the focus should shift to other potential solutions such as alternative estimation methods (A. A. Withagen‐Koster, R. C. van Kleef, & F. Eijkenaar, To be submitted for publication),^3^, ^39^ sophisticated forms of ex post risk‐sharing,^37^, ^40^ and relaxing premium regulation.

In conclusion, the performance of the Dutch RE model can be improved by adding an indicator for “being healthy” based on multiple‐year low spending. Irrespective of which spending threshold is ultimately used, however, risk‐selection potential remains. Given that risk selection is highly undesirable, further improvement of RE merits high priority.

## CONFLICT OF INTERESTS

All authors declare there are no conflict of interests.
